# Adults Experiencing Homelessness in the US–Mexico Border Region: A Photovoice Project

**DOI:** 10.3389/fpubh.2017.00113

**Published:** 2017-05-19

**Authors:** Eva Margarita Moya, Silvia M. Chavez-Baray, Jacqueline Loweree, Brian Mattera, Nahomi Martinez

**Affiliations:** ^1^Department of Social Work, College of Health Sciences, University of Texas at El Paso, El Paso, TX, USA; ^2^School of Social Work, Temple University, Philadelphia, PA, USA

**Keywords:** homelessness, photovoice, US–Mexico Border, health, advocacy

## Abstract

Homelessness is a social, economic, and political crisis in the United States. In particular, the US–Mexico Border region has seen a surge of homelessness, specifically among veterans, women victims of intimate partner violence, and immigrants. In 2014, 12 persons in El Paso, TX, with experience of being homeless used the photovoice methodology to participate in a project titled, “The Voices and Images of the Residents of the Opportunity Center for the Homeless: A Visual Project on the Identity and Challenges Homeless Adults Face on the Border Region.” The project was led by faculty from the Department of Social Work and facilitated by graduate students from the Departments of Social Work, Sociology, and Anthropology at the University of Texas at El Paso. In partnership with the Opportunity Center for the Homeless, a community-based organization, a gallery of photographs with respective narratives was produced along with a video documentary. The participants identified four themes: broken systems, invisibility, opportunities and what works, and growth and determination. These themes represent participants’ life experiences with homelessness and their aspirations. In addition to the photo gallery, participants supported the development of a Call to Action asking the community, policy, and decision makers to commit to changing the current social, economic, and political conditions affecting individuals experiencing homelessness. The gallery, Call to Action, and overall participant experiences with photovoice were shared during local, regional, and national conferences and events, including three State of the Homeless Conferences led by the Opportunity Center for the Homeless in partnership with the university.

## Introduction

Many take the concept of “home” for granted. Even those of us who are unsatisfied with our apartments or houses, and who dream of living elsewhere, usually have a place to sleep at night and space to call our own. However, for an alarming number of people ranging from infants to elderly, “home” is an elusive—and perceivably unachievable—dream. These are the populations we refer to in this article as persons experiencing homelessness. Overwhelmingly, people do not choose to be homeless. Homelessness most often occurs as a result of personal challenges and societal failure to address basic human protections. It is an international phenomenon with complex causal mechanisms and implications. Low income, limited education, unemployment, illness, disability, physical and mental health, and substance abuse, as well as experiences with child welfare and the justice system, can be understood as contributing biopsychosocial factors (1–3). Given such complexity, effectively addressing the needs of individuals experiencing homelessness or otherwise inadequate housing requires a nuanced and multifaceted approach.

The definition of homelessness used by the US Department of Housing and Urban Development (HUD) is broad and encompasses many states of precarious living and situational experiences. These include: (1) individuals and families who lack a fixed, regular, and adequate nighttime residence, some of whom may be exiting an emergency shelter or a place not meant for human habitation where they resided for 90 or fewer days; (2) individuals and families who will imminently lose their primary nighttime residence; (3) unaccompanied youth, families with children, and (4) individuals and families who are fleeing—or are attempting to flee—intimate partner violence, sexual assault, stalking, or other dangerous, violent, or life-threatening conditions (4).

The HUD definition is laudable for inclusivity, as evidenced by the range of circumstances and causes of homelessness described previously. Nonetheless, this definition is in and of itself inadequate to address experiences of and social responses to different homeless subgroups. Harmful and marginalizing perceptions portray some—but not all—homeless populations as “lazy,” “alcoholic(s),” “unemployed,” and “crazy,” among others (5, 6).

The diversity of the homeless population and variation in residential stability make it difficult to estimate the incidence and prevalence of homelessness. This challenge is exacerbated by definitional and methodological differences among state and non-profit organizations that measure homelessness (7). National studies conducted in the 1990s found that the lifetime prevalence of homelessness among families in the United States was on the order of 10%. In other words, approximately 1 US household in 10 contained at least one member who had experienced homelessness. According to the 2016 Annual Homeless Assessment Report to Congress, on a single night in 2016 a total of 549,928 individuals were homeless in the United States and residing in either shelters, transitional living centers, “safe havens,” or unsheltered places not intended for inhabitation (8). Children are also profoundly impacted by homelessness. According to the US Department of Education, 1.2 million students enrolled in public schools are without fixed and adequate residences; other estimates suggest that as many as 2 million students experience homelessness each year (9).

Individuals experiencing homelessness need multiple services in order to address the complex effects of inadequate housing, health disparities, and other unmet needs. The underlying root causes of homelessness are grounded in social, political, and economic stratification and include poverty, medical and/or mental health conditions, lack of affordable housing, displacement, food insecurity, substance use conditions, fleeing violence and/or immigration status, unstable employment, inaccessibility to public programs such as education and/or housing, stigma, and discrimination (10–14). Unfortunately, the voices of individuals experiencing homelessness are often excluded from the structural and systemic decisions that impact their lives (6) and inhibit or facilitate their access to community services (12, 15). Homelessness remains a salient challenge across multiple marginalized populations, such as immigrants, LGBTQI persons, and the disabled—especially those who live in remote, rural, and border areas with limited infrastructure and services. Local, state, and federal resources are urgently needed to address the needs of individuals and families, especially those who are for the first-time experiencing homelessness and to mitigate the conditions and risk factors that produce episodes of homelessness (3, 16).

Given the transient nature of individuals living in emergency shelters or on the street, persons experiencing homelessness (PEH) typically do not report having a sense of belonging (i.e., psychological integration) with their communities—a pattern that is consistent across gender, age, and racial/ethnic groups (17). Such social isolation may contribute to the risk of mental health concerns or exacerbate existing conditions. Indeed, homelessness is associated with an increased risk of developing mental disorders, and those who already experience mental disorders in the United States have a 20–50% risk of becoming homeless at some point in their life, approximately 20 times higher than that in the general population (18, 19). In short, homelessness is a complex and open-ended phenomenon. In popular discourse and institutional policy, the issue of homelessness is too often understood through narrow preconceptions that are transcended by real-life experiences (20).

### State of Homelessness in El Paso, TX

Situated at the midpoint of the 2,000-mile US–Mexico border is the dynamic, growing, and binational El Paso and Ciudad Juarez metroplex community of about 2.5 million residents (21). The population of El Paso is 835,593, with 81.3% self-identified Latinos/Hispanics (22).

El Paso and Ciudad Juarez, Mexico share systemic health inequalities and social injustices that transcend the Rio Grande River. This region is characterized by unique cultural, socioeconomic, and environmental factors that complicate the ability of policymakers and health and human service professionals to address public health issues such as homelessness. In El Paso, TX, the unemployment rate is twice the state and national average, and per capita income is one-third the national average. Additionally, 32% of children and 20% of households live below the poverty line, and 31% of adults lack health insurance (23).

A 2016 Point-in-Time survey documented 1,222 persons experiencing homelessness on a single night currently living in El Paso, TX (24). Relative to the general population, El Paso may appear to have an unusually large Hispanic homeless population. However, this may be more associated with data collection and what some have called the “Hispanic Homeless Paradox” rather than actual differences in prevalence (25, 26). According to the HUD (27), the “Hispanic cultural orientation” may explain why so many Hispanics who might otherwise be living on the streets are doubling or tripling up with friends and relatives, often in overcrowded conditions. It is important to note that a large number of Hispanics are marginally housed and are underrepresented in state and federal statistics. Marginally housed Hispanics have traditionally gone unidentified and under researched, due to the difficulties in current methods of gathering data (26).

Along the US–Mexico Border, homelessness is an urgent and binational matter. Persons experiencing homelessness who reside on the US side of the border often travel to Ciudad Juarez to access inexpensive hotels, apartment rooms, clothes, food, illegal substances, alcohol, and sexual services. Border crossings may also facilitate the ability to elude law enforcement, increase day labor opportunities, engage in smuggling and other informal economic activities, seek out culturally distinct forms of empathy and compassion, and enjoy independence and freedoms not available in the country of origin (20).

In 2014, 12 persons in El Paso, TX, with experiences of homelessness took part in a photovoice project entitled, “The Voices and Images of the Residents of the Opportunity Center for the Homeless: A Visual Project on the Identity and Challenges Homeless Adults Face on the Border Region.” Researchers from the departments of social work, sociology, and anthropology at the University of Texas at El Paso led the effort. In partnership with the Opportunity Center (OC), a community-based organization for PEH, participants and researchers produced a gallery of photographs with respective narratives as well as a video documentary. Participants further supported the development of a “Call to Action” asking the community and policymakers to commit to changing the current social, economic, and political realities affecting PEH. The gallery, Call to Action, and overall participant experiences were shared during local, regional, and national conferences and events, including three State of the Homelessness Conferences led by the Opportunity Center in partnership with the University of Texas at El Paso.

### The Opportunity Center for the Homeless and the Photovoice Project

The OC is an agency that advocates, supports, and protects PEH from the perils, stigmas, and exclusion that they face and is situated on the US–Mexico Border region in one of the poorest zip codes in the country (28). The center was established in 1993 and is the largest shelter system in El Paso County. Originally, the shelter was only able to provide individuals with a temporary shelter and coffee during the cold winter months. Presently, the center operates 10 different housing facilities that provide numerous services to a range of individuals including veterans, single women and men, families, individuals with mental illness, and the elderly. All individuals are served regardless of their varying stages of homelessness. The facilities are specialized to meet the housing and service needs of the unique populations the center supports. The Center for the Homeless aligns its structure with the vision of HUD.

The center’s mission is to serve all PEH without distinction of gender, race, ethnic origin, language spoken, or religious beliefs, and regardless of mental, drug, and alcohol problems. The OC seeks to empower PEH to move beyond their condition if they are capable. If this is not possible, as may occur for individuals with developmental disabilities or other barriers, the OC provides other appropriate support and services through case management, referrals, and service coordination (29).

Persons in different stages of homelessness are mostly supported through one of these major housing programs, as defined by HUD: emergency, transitional, and permanent housing. During the emergency phase, residents are allowed a temporary stay and share living quarters with all other residents. Transitional homes are designed to house individuals for longer periods of time. These programs offer more reliable and private living spaces. Residents can stay for up to 2 years and are provided their own bed in an open space shared by a smaller group. At one point, HUD supported homeless shelters with funds for social services; however, given policy changes at the federal level, support for these services has dwindled as HUD has shifted greater focus toward Housing-First (the approach that provides PEH permanent housing first followed by services as needed), and Wrap-around Services Programs (programs that specialize in non-housing services such as education, job training, and health care) (30) during the last several years.

The OC operates one permanent supportive housing facility that provides residents with subsidized private apartments and case management assistance. Additional resources include a safe haven and the single room occupancy shelter. Residents are each provided their own private room and share a kitchen and bathroom. While these are also temporary shelters, the precise length of each resident’s stay depends on their personal situation, including mental health status and age. What distinguishes the OC from other homeless shelters in the country is that, aside from housing services, it also provides an array of wrap-around services onsite. Residents from any of the 10 facilities can access medical, mental health, nutritional, educational, and career counseling services. The center’s diverse residents, mission to protect homeless persons, and established roots in the community created an ideal opportunity to engage residents in a photovoice project.

The photovoice approach is a participatory action research method aimed at creating meaningful conversations and generating action around a social issue (31). Photovoice is distinguished from conventional research methods in that the method allows the participants to engage actively throughout the entire research process with the guidance of trained researchers. The primary goals of photovoice are to enable participants to record and reflect on their community’s strengths and concerns, promote critical dialog and knowledge about important issues, and to reach policy and other key decision makers (32, 33). The technique is based on the understanding that if policies and decisions are derived from the integration of local knowledge, skills, and resources within affected populations, the policies will be more effective (31). Some theoretical underpinnings used to inspire photovoice include education for critical consciousness, feminist theory, action research, and non-traditional approaches to documentary photography (34).

This method has been the topic of well over 100 research articles in sociology, social work, public health, anthropology, and nursing (34); and most photovoice endeavors employ a participatory action research strategy with small samples (e.g., 5–16 participants) with an emphasis on qualitative approaches (31). Photovoice has also been used as a community assessment tool to describe homelessness in Ann Arbor, MI, USA (35).

### Project Overview

The “Voices and Images of Homelessness” project adopted the following aims: (1) empower and mobilize PEH to gain access to policymakers and decision makers; (2) promote critical thinking through formative research that helps create effective communication strategies for advocacy; and (3) portray everyday life realities of homelessness in the border region using personal and human perspectives. Research questions included (a) What are the characteristics of adults experiencing homelessness in El Paso? (b) How does homelessness affect participants’ mental and physical health? (c) How do social stigmas affect participants’ quality of life? (d) What are some of the barriers to accessing health and human services within the region? and (e) How do PEH in the border region deal with their challenges? The project protocol received Institutional Review Board approval from the University of Texas at El Paso in 2014.

## Materials and Methods

The study was conducted at the OC between August 2014 and February 2015. A total of 12 persons (*n* = 12) participated in the study. To be eligible for inclusion, individuals had to be age 18 or older, report a history of homelessness, and reside in one of the OC shelters. Participants were recruited by shelter managers based on the inclusion criteria. Participation was voluntary, and no economic stipend was provided. The team presented participants with weekly tokens of appreciation, such as hygiene products, care packages, and notebooks for personal use. Participants asked that their names be used to identify their stories; no pseudonyms were used. The study was carried out in accordance with the recommendations of the University of Texas at El Paso Institutional Review Board. The protocol was approved by the University IRB Committee.

### Procedures

Once the participants were recruited, the following protocol was implemented (36): (1) written consent of participants was secured; (2) an orientation and ethics training was conducted; (3) participants took photos; (4) participants and researchers convened in weekly meetings to discuss the photos; (5) steps 2–3 were repeated as needed; and (6) an exhibit to present results and issue a Call to Action (a summary of the problem and a list of actionable steps the public can take to address the problem) to decision and policymakers was developed. The project ultimately included a total of five group meetings including one orientation session and four photo sessions.

The research team, composed of faculty and graduate students from sociology and social work, conducted a 2-hour orientation and ethics training during the first session. Representatives from the OC were invited to attend to build capacity in participatory action research. The orientation covered the project’s methodology, goals and objectives, and ethical concerns. Team members conveyed the power and ethics of taking photographs for public use and display and instructed study participants to take proper precautions when photographing themselves or others. The importance of obtaining consent of individuals in all photographs was communicated. Consent forms in English and Spanish were provided to the project participants to present to persons they wished to photograph.

The group agreed to attend weekly meetings at the OC. All participants signed informed consent forms. At the end of each of session, participants were provided with disposable cameras and a series of “framing questions” to keep in mind when taking photos for the project. They were provided a “SHOWeD” form to answer the following questions for each image: What do you *S*ee here? What is really *H*appening here? How does this relate to *O*ur lives? *W*hy does this problem or situation *e*xist? What can we *D*o about the problem or situation? These questions were used to probe for deeper understanding during the individuals’ sharing of photos. Participants were asked to carry consent forms and cameras with them for a period of 4–5 weeks and to shoot one or two rolls per week. After orientation, each participant was asked to select two or three photos to share with the group at subsequent meetings.

Participants collectively identified images that made the greatest impression on them. A total of 32 photographs and narratives, drawn from an initial pool of 400, were selected by them to be part of the project’s gallery. Study participants were actively included in drafting the Call to Action (see subsequently). In order to gain more in-depth insights about the lives of the OC residents, all participants were asked to be part of a one-on-one audio and video short interview session. This culminated in a 20-min documentary presenting the project findings for general audiences.

### Analysis

The participants engaged in a three-stage process of participatory analysis facilitated by the university faculty and graduate students based on Wang and Burris (32). Analysis was facilitated by (1) selecting the photographs that most accurately reflected their concerns and perspectives on homelessness; (2) contextualizing (telling stories) about what the photographs meant to them; and (3) identifying emergent themes. Although some quantitative data were collected from the participants, the majority of the data were compiled qualitatively in the form of 32 photo images and individual narratives (collected from the SHOWeD forms), session notes, debrief reports, and session audio recordings. Using content analysis (a qualitative analytical approach that identifies surging themes and categorizes data) (37) was performed to classify these 32 photos into themes.

Participant demographics included age, gender, employment status, education level, and ethnicity. Photos and accompanying stories selected for discussion were posted on the wall for viewing. After three sessions of discussing photographs related to social issues and 2 weeks of relating these challenges to experiences of homelessness, participants voted on which photographs and stories captured the most essential themes.

## Results

**Figure 1:**
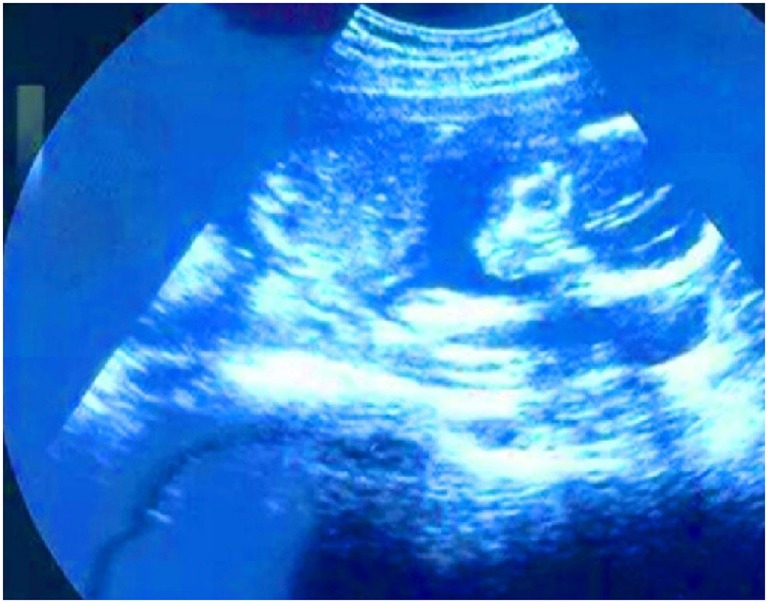
**A new life**. I see my son at 5 months and 7 days. I couldn’t get checked for the first 4 months because I didn’t have an ID. After the fourth month, one of my sisters looked for me to try to help me. I got checked for the first time in the fifth month. The gynecologist told me everything was fine. After seven days I started to feel pain. The cervix had torn and the baby came out, it was as if I were giving birth. It hurts even today. Many homeless women lose opportunities to receive timely and needed health and social services. We need access to health and medical care (Judith).

**Figure 2:**
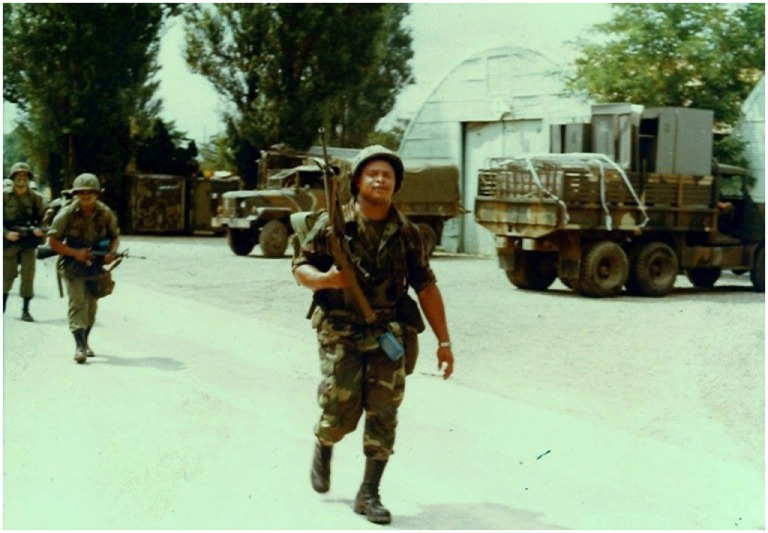
**Broken promises**. I was discharged from the Army unjustly. I did not have the chance to voice my opinion to the Discharge Review Board. I passed all my Skilled Qualification Tests, I made it to Rank E-5, and had plenty of letters of recommendation. I believe I should have been discharged with some type of disability, since the reason I was discharged was because my test scores were too low. I went to school for 4 weeks while in basic training for math, reading comprehension, and I was never told I was not qualified to be in the military. I re-enlisted for another 4 years without being told that I was not qualified to be in the military. I believe this was done because it was still the Vietnam Era. I feel that if it was wartime, if my scores would have made a difference. I wanted to retire at 20 years and I felt that was stripped from me. Because Vietnam was no longer a priority, the military didn’t need us any longer (Ricky).

**Figure 3:**
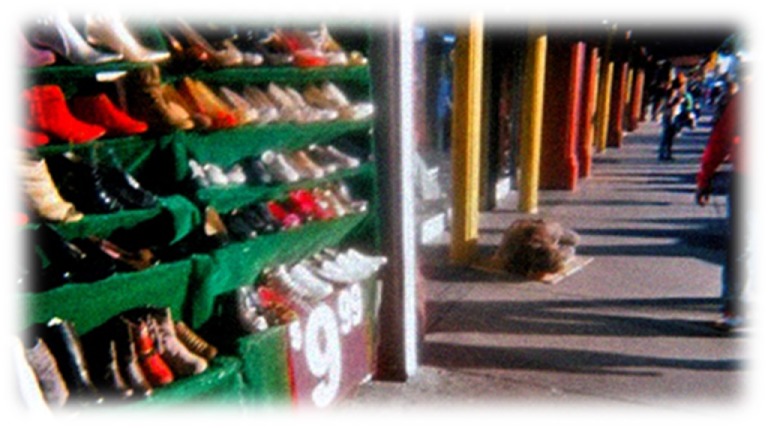
**From invisibility to inclusion**. I saw a man who put a cardboard down on the ground and lay down to sleep. People walked by, looked down and shook their heads. They acted like this guy was trash. Some people fall through the cracks. We need to educate each other about the reasons why people become homeless and provide resources to those that are vulnerable before they become homeless. The lack of resources and opportunities force people to end up in the street. Inform yourself about the services that exist. That way people can try to get back on their feet (Nancy).

**Figure 4:**
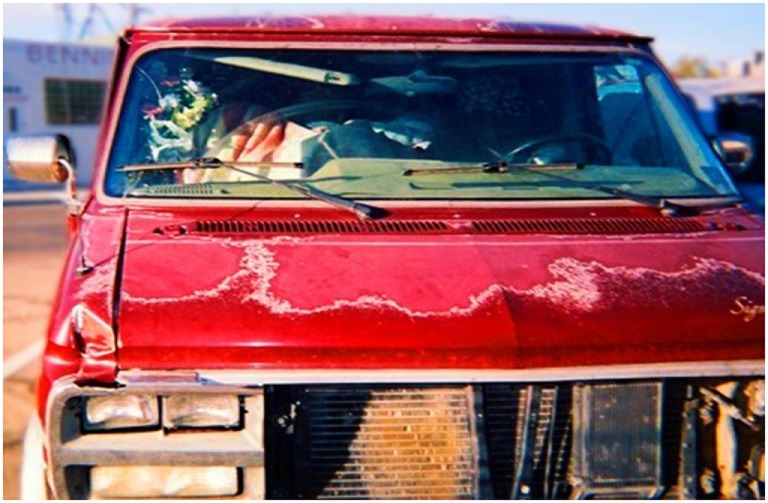
**Living**. I see a van full of clothes, I see a way of life. Someone is living in a van. It can happen to anyone. Things happen in our lives that are uncontrollable. Help one another and educate each other on the causes behind homelessness (Annette).

**Figure 5:**
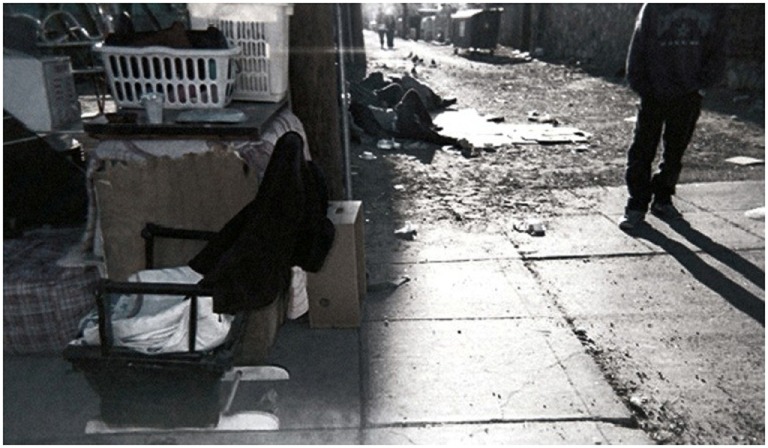
**Investments**. I see people trying to survive. There is nowhere to go. Homelessness is everywhere. It is a reality. We need community awareness and action to address homelessness. Help one another. Each one teach one. Governments should ensure federal, state and local funds to support positive efforts to end homelessness (Annette).

**Figure 6:**
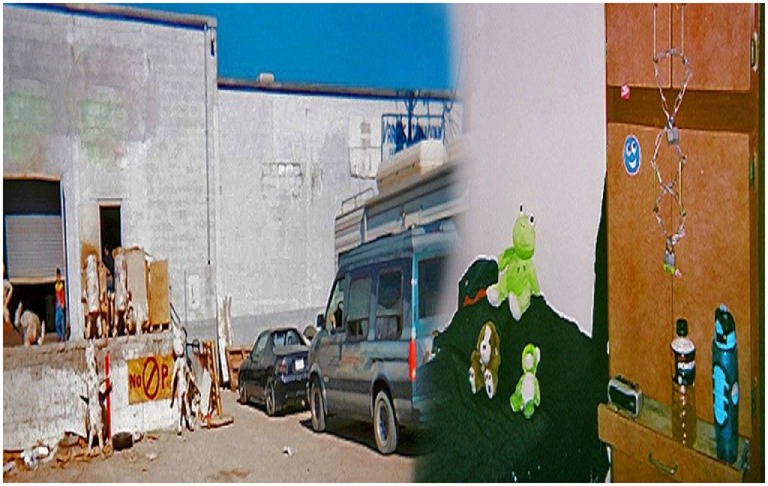
**Before and after**. Piñatas, a car, a van, and empty space with trash around. I lived in here for three years. I slept in one of the rooms and worked making piñatas. It’s a dirty place. We work without papers and there are really no opportunities. We have no rights nor privileges. There is abuse … we live in hiding. This situation needs to change. My new home is now the Women’s Transition Living Center (TLC) at the Opportunity Center. I have four companions. Luis, who helps me to defend myself, Bondo, helps me to say no to drugs, Joanna is there when I need her the most, and Julia wipes my tears off. Sometimes family shuts you out because you are illegal. I’m moving forward with my new life. I have an education and a place to come home. My work situation has not changed, I am determined to change it (Judith).

**Figure 7:**
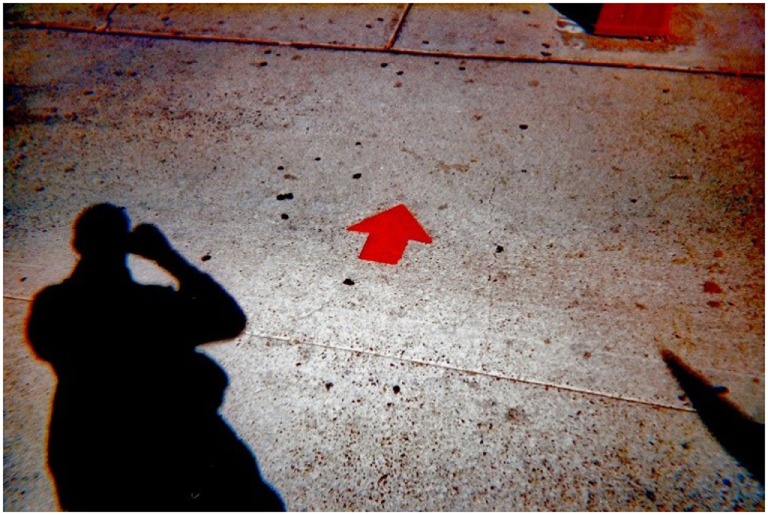
**Homelessness can happen to anyone**. I have I have a College degree from Colorado and a history of 10 years in the military. After I left the military, I ended up doing a lot of minimum wage jobs and ended up being homeless. I stayed in hotels in Ciudad Juarez, outside the bus station, at restaurants until I found the Opportunity Center through the internet. These minimum wage jobs were eating my life little by little. I am now working and living at the Veterans Transition Living Center (VTLC). I have a son and he is my motivation. I want to save some money and to go back to school and I want to take care of my son. Don’t be afraid of us. We are not bad people. With direction, support services and guidance we can overcome homelessness (Stephen).

**Figure 8:**
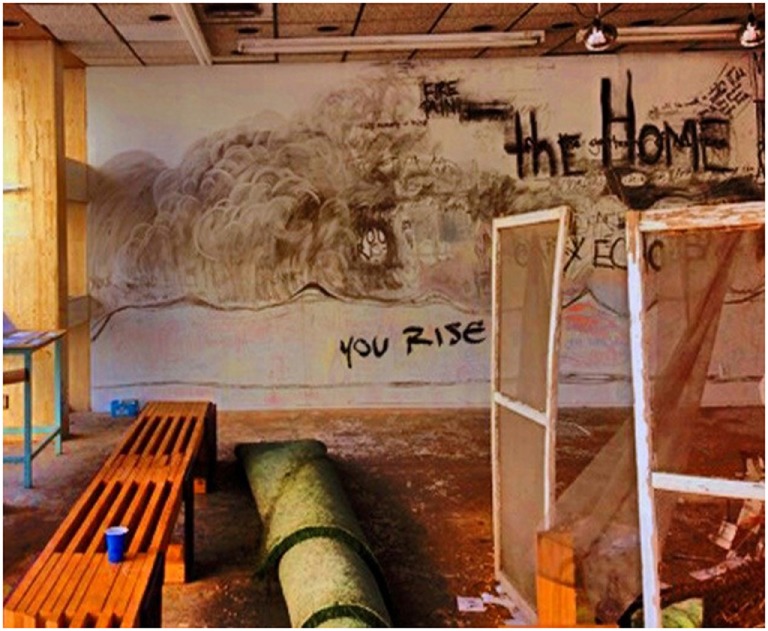
**You rise**. As I was walking, I found myself looking through the windows of old vintage buildings. I stumbled across words that said peeked in, I realized that someone had built a façade of a home. It had windows, grass, a bench, a desk and some lights. When I looked closer at the wall with the writing, I almost lost my breath when I read the words “You rise” in the midst of the dark shadowing. I remembered my time as a teenager, jumping from house to house and hoping that I could bring myself out of homelessness. I see myself now, in my last semester of graduate school, and being humbled and molded by my experience. This photo represents hope, confidence and determination in releasing ourselves from the chains that bring us to homelessness (Courtney).

Participants grouped photographs and accompanying stories into four categories that emerged from the discussion and participatory analysis: *Broken Systems, Invisibility, Opportunities and What Works*, and *Growth and Determination*. These themes resulted in the Call to Action. The gallery was organized using the thematic narratives to describe overall experiences of homelessness. The following sections summarize participant characteristics and demographics along with each theme and present examples of the narratives associated with topics (see Figures 1–8).

### Participant Characteristics and Demographics

Participants were asked about their education and employment histories along with their reasons for residing at the OC. Of the project, eight participants identified as female (*n* = 8) and four as males (*n* = 4). In terms of ethnicity, more than half of the participants self-identified as Hispanic (*n* = 7), four as White (*n* = 4) and one as African American (*n* = 1). The mean age was 41 years, with participants ranging from 23 to 59 years of age. There was an equal distribution among those with less than a high school diploma (*n* = 4), a GED or high school diploma (*n* = 4), and a college degree (*n* = 4; two participants were enrolled in a graduate program). Seven reported being unemployed, four (*n* = 4) identified as disabled and three (*n* = 3) were veterans. All women in the study (*n* = 7) reported a history of intimate partner violence prior to experiencing homelessness. Reasons for residing in the OC included losing a family, history of substance abuse, and intimate partner violence.

### Broken Systems

One of the most pertinent points of discussion during the photovoice sessions were “Broken Systems or Services” and the external challenges involved with experiencing homelessness in systems that failed to secure basic human needs (e.g., access to health care, mental health services, education, and safety). Through their narratives, participants shared feelings of frustration, incompetency, and betrayal when attempting to navigate health care clinics, the military, immigration services, and agencies tasked with protecting youth.

One of the most moving narratives was the story of Judith. In her photo titled “A New Life” (Figure 1), Judith bravely shared her struggle with a pregnancy that resulted in miscarriage due to her inability to access timely pre-natal care. Immigration status and fear of deportation had posed tremendous barriers.

I see my son at five months and seven days [into the pregnancy]. I couldn’t get checked for the first four months because I didn’t have an ID. After the fourth month, one of my sisters looked for me to try to help. I got checked for the first time in the fifth month. The gynecologist told me everything was fine. After seven days, I started to feel pain. The cervix had torn and the baby came out; it was as if I were giving birth. It hurts even today. Many homeless women lose opportunities to receive timely and needed health and social services. We need access to health and medical care.

Judith

Participants shared numerous stories of physical and mental health conditions that were exacerbated by barriers to care. Limited or non-existent financial resources, insurance status, limited knowledge about how to obtain care, difficulties purchasing medications, and lack of transportation were among the most common barriers.

Participants reported a lack of support in navigating complex health systems and expressed major concerns with access to medical care and follow-up appointments. Several participants also reported inconsistencies in care and referred to use of emergency rooms and urgent centers as their primary source of services. Their experiences reflected a broader issue facing homeless populations: many PEH are discharged from hospitals and clinics back to the streets or to homeless shelters, which significantly compromises their recovery (38). Multiple participants reported going years without receiving medical, behavioral, or recovery services. In addition, many expressed confusion about the process of navigating the health and human services systems and shared overall misconceptions of these systems, expressing negative opinions and structural barriers that reduce the likelihood of PEH engaging in and maintaining treatment in behavioral health services.

One of the veterans, Ricky, described feeling used by the military and discarded unjustly when he was no longer needed. Ricky’s disbelief and disillusionment with a system that he had faithfully dedicated years of his life to was very clear when he described his photo “Broken Promises” (Figure 2). Although he does not blame the military for his homelessness, he stated feeling “stripped” of what could have been a prosperous career.

I was discharged from the Army unjustly. I did not have the chance to voice my opinion to the Discharge Review Board. I passed all my Skilled Qualification Tests. I made it to Rank E-5, and had plenty of letters of recommendation. I believe I should have been discharged with some type of disability, since the reason I was discharged was because my test scores were too low. I went to school for four weeks while in basic training for math, reading comprehension, and I was never told I was not qualified to be in the military. I re-enlisted for another four years without being told that I was not qualified to be in the military. I believe this was done because it was still the Vietnam Era. I felt that if it were wartime, my test scores would not have made a difference. I wanted to retire at 20 years of service and I felt that was stripped from me. Because Vietnam was no longer a priority, the military didn’t need us any longer.

Ricky

Ricky’s story reflected the experiences of the other veterans in the group. The three veteran participants reported barriers to services as substantial, and indicated that services exist in a fragmented manner. Through his experience residing at the OC, he indicated finding inner strength. Trust and support were considered important to participants for overcoming adversity and homelessness, achieving self-sufficiency and personal goals.

### Invisibility

Participants described feeling invisible, both within the fragmented and broken systems they navigated and their own communities. Homelessness is everywhere, but it is a problem many do not acknowledge. Nancy captured the phenomenon of public indifference to homelessness in her photo, “From Invisibility to Inclusion” (Figure 3), in which she advocates for greater education on the root causes of homelessness and resources to address mental and health care needs.

I saw a man who put a cardboard down on the ground and lay down to sleep. People walked by, looked down, and shook their heads. They acted like this guy was trash. Some people fall through the cracks. We need to educate each other about the reasons why people become homeless and provide resources to those that are vulnerable before they become homeless. The lack of resources and opportunities force people to end up in the street. Inform yourself about the services that exist. That way people can try to get back on their feet.

Nancy

In the photograph, “Living” (Figure 4), Annette captured the experience of a person living in a van.

I see a van full of clothes; I see a way of life. Someone is living in a van. It can happen to anyone. Things happen in our lives that are uncontrollable. Help one another and educate each other on the causes behind homelessness.

Annette

Participants described feeling lonely and invisible, as well as having broken relationships with their families and society. They discussed feeling stigmatized by employers, family, and community members. Self-stigma and social stigmas were identified by most of the participants as barriers to recovery and reintegration.

### Opportunities and What Works

Despite their struggles in experiencing homeless, participants found some opportunities and support. Annette’s photo, “Investments” (Figure 5) conveys her “each one teach one” philosophy, advocating for the shared responsibility between PEH and those who serve vulnerable populations.

I see people trying to survive. There is nowhere to go. Homelessness is everywhere. It is a reality. We need community awareness and action to address homelessness. Help one another. Each one teach one. Governments should ensure federal, state, and local funds to support positive efforts to end homelessness.

Annette

Participants described how their quality of life and overall well-being deteriorates after they become homeless. Participants shared that what works in helping them cope with homelessness is not just seizing opportunities, but also receiving help from the support structures that advocate for housing, education, health and mental health care, employment, and legal rights.

From Judith’s photo, “Before and After” (Figure 6), we learned how having a safe home at the Opportunity Center’s Transitional Living Center improved her life:

I see piñatas, a car, a van, and empty space with trash around. I lived here for three years. I slept in one of the rooms and worked making piñatas. It’s a dirty place. We work without papers and there are really no opportunities. We have no rights or privileges. There is abuse … we live in hiding. This situation needs to change. My home is now the Women’s Transitional Living Center at the OC. I have four companions (stuffed animals). Luis, who helps me defend myself, Bondo, helps me say no drugs, Joanna is there when I need her the most, and Julia wipes tears off. Sometimes family shuts you out because you are illegal. I’m moving forward with my new life. I have an education and a place to call home. My work situation has not changed. I am determined to change it.

Judith

The themes of support, services, positive changes, and stable employment were prominent in photographs and stories. Participants discussed the difficulties they encountered securing jobs due to stigma, discrimination, lack of permanent residence, immigration status, legal issues, addictions, and mental distress. Those who found work sometimes struggled to connect with employers and coworkers. However, there were also positive and hopeful stories. Some individuals recalled choosing to return to school. Some drew motivation from peers, children and family, or supportive shelter staff.

### Growth and Determination

The fourth theme, “Growth and Determination,” emphasized what participants had learned and what they wanted to become. Stephen shared that homelessness does not discriminate, and that his son is his motivation, in “Homelessness can Happen to Anyone” (Figure 7).

I have a college degree from Colorado and history of 10 years in the military. After I left the military, I ended up doing a lot of minimum wage jobs and ended up being homeless. I stayed in hotels in Ciudad Juarez (Mexico), outside the bus station, at restaurants until I found the Opportunity Center through the internet. These minimum wage jobs were eating my life little by little. I am now working and living at the Veterans Transition Living Center. I have a son and he is my motivation. I want to save some money and go back to school and I want to take care of my son. Don’t be afraid of us. We are not bad people. With direction, support services and guidance, we can overcome homelessness.

Stephen

The participants also expressed that this theme was characterized by learning new skills, traits, and things about themselves, especially in the context of education and employment. For example, participants discussed their desire to pursue higher education and secure a job. They recognized that in order to achieve their goals, they might have to change.

PEH emphasized how homelessness can result in a sense of isolation and distrust of others due to the perceived or real disruption of social connections, physical separation from loved ones, and inability or unwillingness to fulfill social role obligations. Feelings of helplessness, loss of control, and traumatic experiences contributed to negative outcomes for many participants. For some participants, reflecting on past struggles was essential for moving forward. Courtney demonstrated this through her photo, “Rise” (Figure 8):

As I was walking, I found myself looking through the windows of old vintage buildings. I stumbled across words that said “peeking in.” I realized that someone had built a façade of a home. It had windows, grass, a bench, a desk, and some lights. When I looked closer at the wall with writing, I almost lost my breath when I read the words “You Rise” in the midst of the dark shadowing. I remembered my time as a teenager, jumping from house to house and hoping that I could bring myself out of homelessness. I see myself now, in my last semester of graduate school, and being humbled and molded by my experience. This photo represents hope, confidence and determination in releasing ourselves from the chains that bring us to homelessness.

Courtney

Participants highlighted the importance of prevention, early intervention, and fostering school readiness and family support at a young age to prevent homelessness. Social support, school readiness, and community connectedness emerged as factors that enhance determination, hopefulness, and resilience in youth and adults who are at risk for academic, emotional, and behavioral problems.

### In Summary: A Call to Action

Participants and researchers collaborated to create a Call to Action. Drawing on themes from the photovoice project, the participants prepared the Call to Action (verbatim) and highlighted a need for collective action about their experiences around:

(1) Increasing the visibility of people affected by or dealing with homelessness through their lives, concerns, vulnerabilities, and aspirations to raise awareness.
(2) Working for inclusion, equality, and participation of persons experiencing homelessness.
(3) Raising awareness about homeless issues among policymakers and decision makers, opinion leaders, and the general community.
(4) Creating prevention and care services for persons experiencing homelessness and integrating them in every setting, from housing to services for mental, physical, legal, and emotional well-being.
(5) Launching strategies and services needed and/or used by diverse homeless populations, including children, youth, adults, elderly, and families.
(6) Creating sustainable and permanent funding sources for education, medical, transportation, employment services and interventions for persons who are homeless or in danger of becoming homeless.
(7) Providing timely and quality access to mental and other health care aimed at preventing and addressing issues that cause people to become chronically homeless.
(8) Educating and empowering persons experiencing homelessness by providing comprehensive resource guides.

### Social Action

Project participants presented the gallery, Call to Action, and documentary to policymakers and decision makers at various local, state, and national forums, including the state capitol, and asked for public commitments to direct efforts to preventing and addressing issues that contribute to end homelessness. In addition, the University hosted a project gallery for one month on campus and produced two feature project stories. The Voice and Images documentary is also used as an educational and training resource for social work students in policy and macro practice courses. The director of the OC uses the gallery and documentary to raise awareness and educate the community about the experiences of homelessness in the US–Mexico Border region. The documentary is available in the OC webpage (http://www.homelessopportunitycenter.org/). In addition, the project gallery and documentary informed and supported the OC planning for launching the first ever “State of the Homelessness Conferences” series in partnership with the University of Texas at El Paso.

## Discussion

Through their photographs, narratives, and analyses, participants illustrated their experiences of homelessness, such as feeling stigmatized, navigating fragmented health and human services, taking on risks, and building their inner resiliency and self-determination to move forward. The themes identified by the participants show that PEH possess multiple challenges and strengths. The narratives of the individuals with diverse experiences that emerged through the photovoice methodology highlight that the pathway to homelessness is not a single or linear cause, and therefore requires a person-in-environment framework (3). Following this perspective, this phenomenon must be conceptualized within the environmental context of the individual experiencing homelessness in order to respond to the individual’s unique psychosocial and contextual factors that resulted in being homeless initially, and may continue to pose barriers to overcoming this condition. To truly comprehend homelessness, it is necessary to include—and even prioritize—the narratives of individuals with diverse experiences (39). Given this crucial need to elicit such personal experiences on an idiosyncratic level, methodologies such as photovoice are salient in working with vulnerable populations.

The project participants perceived pervasive stigma and experienced barriers to health and mental care. Lack of consistency in service providers and discontinuity of care are central issues affecting homeless individuals and families (40). Frequent use of the emergency department for care and in patient hospitalization was cited by some of the participants. This frequent use of health services is also widespread among the general homeless population. For example, persons experiencing homelessness are three and four times more likely than those of the general population to use emergency room services for medical care, respectively (41). The rates of primary care engagement among PEH are low, while non-adherence to prescription medications, primary, and secondary care is high, in comparison to the general population. That is why it is so important that policies and programs focus on evidence-based practices and ensure support and resources for wrap around services to prevent and address PEH. Creating environments that promote protective factors and foundational skills can produce a cascade of positive effects.

Several actions on the part of policymakers and service providers may contribute to engendering positive health outcomes among PEH, including securing housing stability, reducing utilization of acute hospital services, educating PEH or at-risk populations on how to engage and advocate for their health needs, controlling costs of care, and mitigating stigma and discriminatory behavior by care providers (42, 43).

The structural barriers that contribute to limited or lack of access to care include low availability or utilization of health insurance, high poverty, rising costs of care, lack of transportation, and limited or inadequate sources of health and mental care (43). A potential solution according to the American Hospital Association (44) would be to ensure that preventative care programs focusing on homeless populations that place and ensure greater focus on increasing continuity of care.

Cultural values in Hispanic and Latino cultures such as “*orgullo*” (pride), “*familismo*” (familism), “*compadrazgo*” (fellowship), and solidarity may influence Latino individuals’ or families’ perceptions when they share their homes, couches, and assets with an individual or who does not have a stable residence (45).

Participants described how services for veterans are limited and frequently fragmented. The veteran participants recommended improvements in outreach and community support and expressed the need for organizations such as the Department of Veterans Affairs (VA) to focus on more timely and effective delivery of services. In order to effectively address multiple, complex needs among veterans and other vulnerable subgroups, providers must hold a comprehensive understanding of the factors and barriers specific to this population that influence health and well-being. The experiences shared by veterans in this project suggest that the current available services for veterans experiencing homelessness are often insufficient and impeded by considerable fragmentation of services. Comprehensive, integrated efforts at the individual, agency, and community levels are needed to address the multitude of factors contributing to and influencing the experience of homelessness among this population, including, but not limited to social exclusion, depletion and lack of social networks, trauma, comorbid mental health and substance use conditions, prior incarcerations, and other marginalized statuses (3, 12, 15).

The critical messages of invisibility and broken relationships with significant persons and professionals were prominent in the narratives and photographs. Support, guidance, and the need to maintain positive relationships with peers were important to the participants. Relationships enhanced participants’ resiliency and determination to see beyond their experiences with homelessness, providing a positive outlook on life and opportunities to strengthen peer support, empathy, and their capacity to overcome their daily challenges. The erosion of psychological well-being among PEH creates difficulties in maintaining significant supportive relationships (46).

The importance of participants identifying the need to socialize with others and seek social support speaks to their awareness and need for factors such as education, employment, resources, self-care, and spirituality. This reciprocal support among people experiencing or with past experience of homelessness encouraged participants to empower themselves, be open-minded, remain sober, and self-determined. Positive caring relationships can nurture resilience, while lack of resilience has been significantly related to hopelessness, loneliness, life-threatening behaviors, and lack of connectedness (47–50). Individuals who perceived themselves as resilient, although disconnected from other people, were less lonely, less hopeless, and engaged in fewer life-threatening behaviors than those who did not perceive themselves as resilient (51). These findings may indicate that interventions promoting health and well-being among this population should emphasize and cultivate the development of positive social support networks to increase resilience and self-empowerment among PEH.

While permanent stable housing was identified by the participants as valuable, they also discussed the need for supportive services. It is not sufficient to simply provide a residence for PEH. To ensure housing stability, services that address other factors contributing to homelessness and housing instability must be integrated, including mental health services, substance abuse treatment, programs to ensure food security, employment services, and long-term medical care (52–57). Wrap-around services that include access to mental health services, health care, and substance abuse care are imperative to the success of residents in permanent supportive housing projects to decrease hospitalization, incarceration, and other social costs (58). Fostering trust in service providers among PEH using person-centered approaches, strengthening the service infrastructure, and training the workforce to serve them are crucial (59). Studies on the pathways that contribute to homelessness such as childhood trauma, mental illness, substance use and addictions, institutionalization, and lack of social networks in border populations and rural areas are needed.

There are several limitations that must be considered when interpreting the results of this study. Generalizability is limited, given the small sample size (*n* = 12) and the fact that all participants were recruited through a single community-based organization. The findings may not be transferable to understanding the lives of individuals experiencing homelessness in the El Paso, TX, area and other US–Mexico border regions.

## Conclusion

To our knowledge, this is the first study on homelessness using the photovoice method in the US–Mexico Border region. Using photographs and narratives, 12 individuals who experience or had experienced homelessness presented alternatives to the negative stigmas frequently attributed to them. The diverse ethnic and cultural backgrounds of the participants offered us the opportunity to learn about diverse perspectives, expressions and experiences of being homeless in the US–Mexico Border region. This study illustrates how many persons experiencing homelessness are conscious of their reality and wish to change their condition. The development of evidenced-based practices to address the needs of PEH and the conditions influencing homelessness require local, state, federal, and binational resources to reduce social and financial costs and improve quality of life.

## Ethics Statement

This study was carried out in accordance with the recommendations of the University of Texas at El Paso, Institutional Review Board committee’ with written informed consent from all subjects. All subjects gave written informed consent in accordance with the Declaration of Helsinki. The protocol was approved by the Unievrsity of Texas at El Paso IRB committee.

## Author Contributions

EM—primary contribution to designing the work, acquisition of information, drafted and revised the work, approved final version, and agreed to be accountable for all aspects of the work. SMCB and JL—substantial contribution to designing, revised it for important intellectual content, approved final version, and agreed to be accountable for all aspects of the work. BM—revised it for important intellectual content, approved final version, and agreed to be accountable for all aspects of the work. NM— substantial contribution to acquisition of information, revised it for important intellectual content, approved final version, and agreed to be accountable for all aspects of the work.

## Conflict of Interest Statement

The authors declare that the research was conducted in the absence of any commercial or financial relationships that could be construed as a potential conflict of interest.
